# A Severe Case of Nuclear Matrix Protein-2-Positive Dermatomyositis With Negative Malignancy Workup

**DOI:** 10.7759/cureus.79178

**Published:** 2025-02-17

**Authors:** Shreya Kondle, Stanley Cohen

**Affiliations:** 1 Internal Medicine, Texas Health Presbyterian Hospital of Dallas, Dallas, USA; 2 Internal Medicine, The University of Texas Southwestern Medical School, Dallas, USA

**Keywords:** ivig therapy, nxp-2, progressive dysphagia, rituximab therapy, s: dermatomyositis

## Abstract

Dermatomyositis (DM) is an immune-mediated inflammatory myopathy that can present with proximal muscle weakness and characteristic skin findings. Nuclear matrix protein 2 (NXP-2)-positive DM, while rare, has a strong association with malignancy. It can display features such as muscle weakness, subcutaneous edema, and refractory dysphagia, all of which were present in our 63-year-old male patient. He experienced a prolonged hospitalization and percutaneous endoscopic gastrostomy (PEG) tube placement. He improved with prednisone, azathioprine, rituximab, and intravenous immunoglobulin (IVIG) treatments. For over two years, he has had no evidence of malignancy. This case underscores the complexity of NXP-2-positive DM and the potential for severe presentations that may require the usage of second-line therapies.

## Introduction

Dermatomyositis (DM) is a rare and acquired immune-mediated inflammatory disease characterized by symmetric proximal muscle weakness, distinctive skin rashes, and potential involvement of other organ systems, such as the lungs, heart, and gastrointestinal tract [1]. Nuclear matrix protein 2 (NXP-2)-positive DM is a rare idiopathic inflammatory myopathy first described in juvenile DM patients and more frequently seen in younger DM cohorts [2]. It has been reported to be associated with a variety of clinical phenotypes, which include severe muscle weakness, joint contractures, polyarthritis, intestinal vasculitis, severe dysphagia, subcutaneous calcinosis, and subcutaneous edema [2-4]. NXP-2 antibody positivity in itself confers an increased risk of malignancy [5].

Our report discusses a course of NXP-2-positive DM with profound weakness and dysphagia that slowly improved with extensive rehab and treatment with rituximab, intravenous immunoglobulin (IVIG), azathioprine, and prednisone. Our patient required prolonged and aggressive treatment, but he displayed a remarkable response to medical therapy. Thus, our patient raises awareness of a rare but potentially severe myopathy that had responded to aggressive treatment. In the literature, there is a stronger association with dysphagia in NXP-2-positive DM patients than in other DM subtypes [6].

## Case presentation

In June 2022, a 63-year-old Caucasian male, with a history of hypertension, hepatitis B, hyperlipidemia without usage of a statin, and type 2 diabetes, presented with three weeks of generalized weakness, with progressive immobility of his lower extremity, bilateral foot pain, and a fluctuating salmon-colored rash around his eyes with periorbital swelling. He worked with heavy machinery, including jackhammers and welding tools, and attributed his weakness to work. He had no history of smoking tobacco. His preliminary labs were notable for elevated alanine transaminase (ALT) at 172 U/L; elevated aspartate transaminase (AST) at 427 U/L; elevated creatine kinase (CK) at 11,119 U/L; normal thyroid-stimulating hormone (TSH) at 3.57 uIU/mL, negative human immunodeficiency virus (HIV) antibodies; and negative hepatitis A, B, and C antibodies (Table 1). He denied fever, unintentional weight loss, night sweats, joint pain or swelling, back pain, photosensitive rashes, Raynaud’s phenomenon, ulcers, dry eyes or dry mouth, vision changes, shortness of breath, cough, chest or abdominal pain, dysphagia, or peripheral neuropathy. On exam, he was afebrile with no rash, nail changes, palpable lymphadenopathy, oral lesions, heart murmurs, focal neurological deficits, abnormal lung sounds, abdominal distention or tenderness, or hepatosplenomegaly. He displayed upper extremity non-pitting edema and grade 4 strength in his lower extremities based on the Medical Research Council (MRC) grading system, which grades muscle power from 0 to 5. He could not raise his bilateral arms above his head.

**Table 1:** Lab values

Test	Patient Value	Reference Range
ALT	172 U/L	4.80-34.20 U/L
AST	427 U/L	0.00-55.20 U/L
CK	11,119 U/L	25-90 U/L
PSA	30.61 ng/mL	0-4 ng/mL

**Figure 1:**
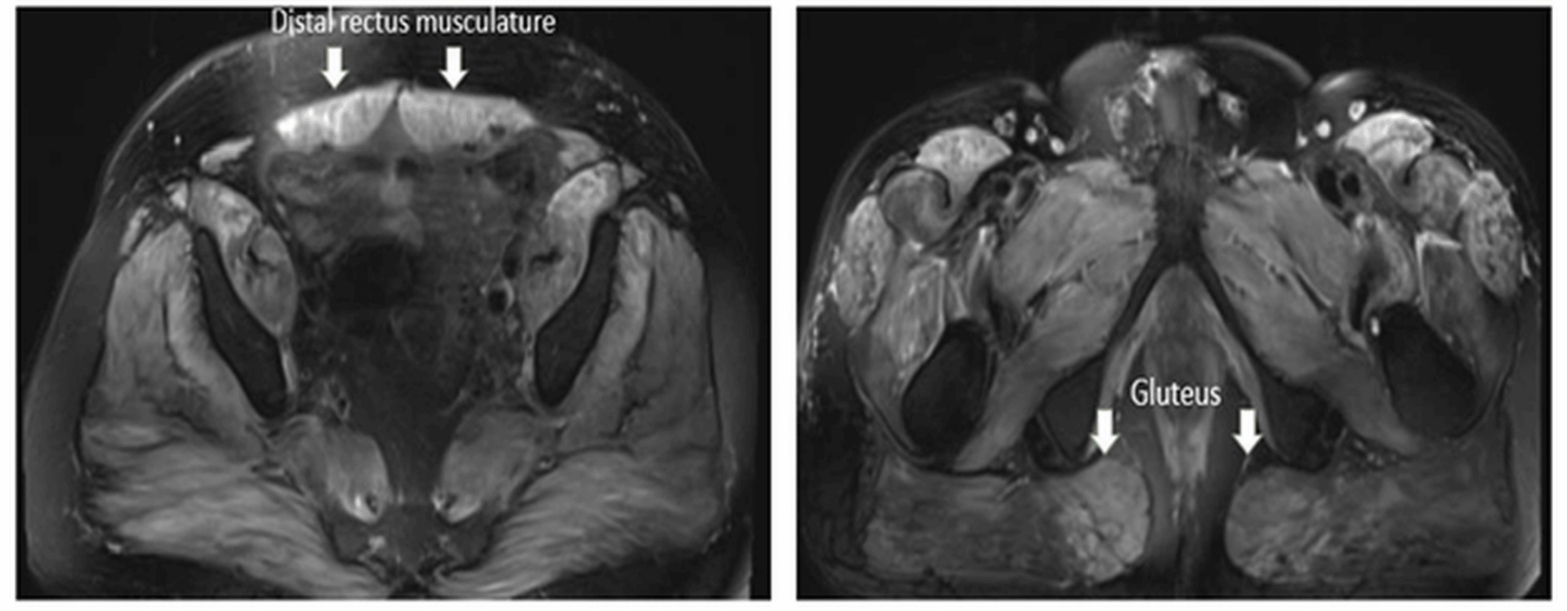
MRI of the lower extremity without contrast - T2 hyperintensity of parapelvic musculature

Liver and abdominal ultrasounds were unremarkable. Initially considered to have rhabdomyolysis, he was treated with intravenous fluids, but his weakness progressed. Thyroid myopathy had been ruled out with normal TSH. With concern for an inflammatory myopathy such as DM, a week later, he received a muscle biopsy of his right quadriceps muscle given his proximal weakness, which revealed no atrophy, inflammatory, vasculitic, or fibrotic changes. This biopsy was taken prior to the initiation of steroids. Later, an MRI of his thigh musculature demonstrated parapelvic inflammatory changes (Figure 1). His weakness continued to progress, and he became wheelchair-bound with severe dysphagia requiring percutaneous endoscopic gastrostomy (PEG) tube placement a month from admission day. At this time, his CK was 20,187 U/L. Further workup revealed normal complement levels (C3, C4), ESR, and CRP. He received extensive testing for myositis-specific antibodies (MSA) and myositis-associated antibodies (MAA). He was ANA positive (1:320) and SSA 52 positive (48 AU/mL). His myositis panel was positive for NXP-2.

**Figure 2:**
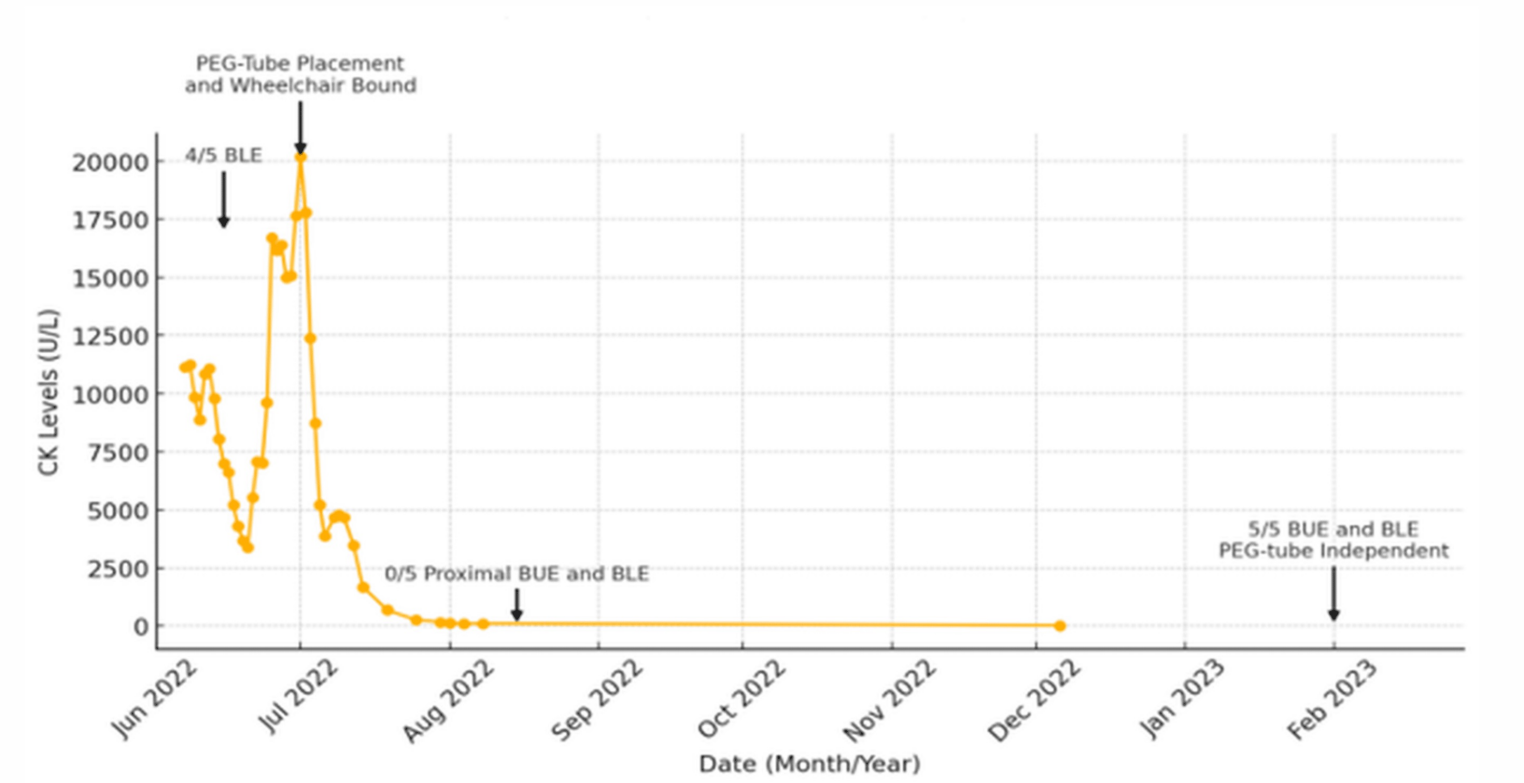
Creatine kinase (CK) levels over time The arrows label the Medical Research Council (MRC) scores of the bilateral upper extremity (BUE) and bilateral lower extremity (BLE), as well as points in time of percutaneous endoscopic gastrostomy (PEG) tube dependence.

Two weeks after admission, he was started on immunosuppressive therapies with IVIG, methylprednisolone, and azathioprine, followed by the addition of rituximab six days later. Altogether, during his two-month hospital admission, he had received two doses of IVIG that were administered as 2 g/kg daily given over two days every 30 days; two doses of 1,000 mg rituximab; three days of 500 mg methylprednisolone daily, followed by 60 mg prednisone with taper by 10 mg every six days; and daily azathioprine. Concurrently, he received sulfamethoxazole and trimethoprim for pneumocystis pneumonia (PCP) prophylaxis and tenofovir alafenamide to cover for possible reactivated hepatitis B given evidence of prior infection with evidence of core antibodies on initial admission. Given the history of prior hepatitis B, myopathy related to hepatitis B was considered but was later ruled out with negative nucleic acid amplification testing one month after initial admission. A malignancy workup was conducted due to the diagnosis of NXP-2-positive DM. Computerized tomography (CT) of his chest, abdomen, and pelvis, upper gastrointestinal endoscopy, colonoscopy, and MRI brain did not reveal malignancy. He had an elevated prostate-specific antigen (PSA) of 30.61 ng/mL that was attributed to his acute DM flare by urology as his PSA decreased through his hospitalization. His CK also normalized at the end of his two-month hospitalization, as shown in Figure 2. While his extremity strength improved, his dysphagia remained unchanged.

He was discharged with a plan to continue IVIG 1 mg/kg on two consecutive days every four weeks, along with azathioprine daily. His dysphagia did not improve while at rehab, and he was readmitted to the hospital for treatment of aspiration pneumonitis two months after his initial admission. At this admission, his CK was normal, and there was no recurrence of his rash or non-pitting edema, but he had continued paresthesias in his legs, which improved with gabapentin and duloxetine. In the outpatient setting, he continues to be followed closely by neurology, rheumatology, and urology with proactive screening for malignancy based on clinical symptoms. Over two years, he has had significant improvement in both his weakness and dysphagia.

In February 2023, he was no longer PEG tube-dependent, and his CK was normal. At this time, he had MRC grade 5 strength in his upper and lower extremities, and he had returned to full-time employment as an electrician. In October 2022, his 10 mg daily prednisone dosage was tapered to reduce the risk of type II fiber atrophy, infection, and worsening bone health, but his weakness worsened, so he remained on 10 mg daily prednisone. In October 2022, electromyography (EMG) was conducted, with no changes in compound muscle action potential, suggesting critical illness neuropathy or myopathy. In October 2023, his IVIG was tapered down to 1 g/kg every four weeks, and his rituximab was delayed due to his health insurance. He had a mild flare, which resolved by increasing IVIG to 2 g/kg every four weeks and receiving a single dose of rituximab. Since then, his symptoms have been stable while he is medically managed with 5 mg prednisone daily, 150 mg azathioprine daily, and IVIG 2 g/kg every four weeks. As of December 2024, he has had no evidence of underlying malignancy, and there are discussions between his neurologist and rheumatologist to taper his maintenance IVIG.

## Discussion

Our patient presented as a severe case of NXP-2-positive DM, which required aggressive treatment with high-dose steroids, rituximab, azathioprine, and IVIG, along with several months of physical and speech therapy for complete resolution of his muscle weakness and refractory dysphagia. His edema was localized to his bilateral upper extremity and was a unique presenting symptom, which is sparsely described in the literature [6]. Our patient had an MRI that revealed inflammatory changes in the parapelvic muscles, such as the psoas and gluteus muscles, but not in the quadriceps where he was biopsied. In the literature, NXP-2-positive DM patients have perivascular inflammation and perifascicular atrophy on muscle biopsy [7]. This type of muscle dysfunction may contribute to severe muscle weakness without significant CK elevations [6]. This is demonstrated in Figure 2, which reveals low MRC scores, indicative of more weakness observed when CK levels were low and vice versa. A repeat muscle biopsy was not conducted to consider other inflammatory myopathies, such as immune-mediated necrotizing myopathy, which can present with severely elevated CK levels, as the patient's progressive dysphagia, lack of statin exposure, and antibody results from myositis panel aligned more with a diagnosis of NXP-2-positive DM.

NXP-2 is a nuclear matrix protein involved in regulating p53-induced cellular senescence [8]. NXP-2-positive DM patients have an increased risk of malignancy compared to the general population when matched by age and sex having an association with malignancy of up to 25% in large cohort studies [6,9]. Malignancies that have been reported include ovarian, lung, breast, non-Hodgkin’s lymphoma, stomach, pancreatic, and colorectal cancers [10,11]. Our patient is a unique case in which no underlying malignant pathology has been found.

NXP-2 antibody positivity has been associated with more refractory DM requiring treatments such as IVIG and rituximab beyond first-line therapies, such as prednisone and disease-modifying drugs (i.e., methotrexate and azathioprine) [11]. Such was the case with our patient who, despite pulse methylprednisolone, subsequent high-dose steroids, and azathioprine, continued to have an active disease with profound weakness and proximal dysphagia requiring a PEG tube. A placebo-controlled randomized trial in 2022 confirmed the benefit of IVIG in DM [12]. Our patient’s response to IVIG and rituximab was slow but dramatic over time to the point that he now works full time as an electrician. In the literature, a delay in treatment initiation and a greater degree of weakness have been related to poorer outcomes in NXP-2-positive DM patients [13].

## Conclusions

NXP-2-positive DM is a rare disease, and our patient's case is notable for the diagnostic difficulties he encountered. His profound weakness, which required extensive rehab and second-line therapies for recovery, also demonstrates a uniquely severe presentation. While NXP-2-positive DM is strongly associated with malignancy, our patient to date has no evidence of malignancy as he continues to be followed closely by neurology, rheumatology, and urology, with proactive screening for malignancy based on clinical symptoms. His positive response to second-line therapies contributes to the literature on the treatment of severe cases of NXP-2-positive DM. He remains without disease activity on 5 mg prednisone daily, azathioprine, and IVIG with plans to taper these treatments.

## References

[1] Qudsiya Z, Waseem M (2023). Dermatomyositis. StatPearls.

[2] Oddis CV, Fertig N, Goel A (1997). Clinical and serological characterization of the anti-MJ antibody in childhood myositis. Arthritis Rheum.

[3] Gunawardena H, Wedderburn LR, Chinoy H (2009). Autoantibodies to a 140-kd protein in juvenile dermatomyositis are associated with calcinosis. Arthritis Rheum.

[4] Espada G, Maldonado Cocco JA, Fertig N, Oddis CV (2009). Clinical and serologic characterization of an Argentine pediatric myositis cohort: identification of a novel autoantibody (anti-MJ) to a 142-kDa protein. J Rheumatol.

[5] Fiorentino DF, Chung LS, Christopher-Stine L (2013). Most patients with cancer-associated dermatomyositis have antibodies to nuclear matrix protein NXP-2 or transcription intermediary factor 1γ. Arthritis Rheum.

[6] Albayda J, Pinal-Fernandez I, Huang W (2017). Antinuclear matrix protein 2 autoantibodies and edema, muscle disease, and malignancy risk in dermatomyositis patients. Arthritis Care Res (Hoboken).

[7] Pinal-Fernandez I, Casciola-Rosen LA, Christopher-Stine L, Corse AM, Mammen AL (2015). The prevalence of individual histopathologic features varies according to autoantibody status in muscle biopsies from dermatomyositis patients. J Rheumatol.

[8] Ueda Y, Shimada K (2023). Nuclear matrix protein 2 antibody-positive dermatomyositis associated with hepatocellular carcinoma. Rheumatol Adv Pract.

[9] Marzęcka M, Niemczyk A, Rudnicka L (2022). Autoantibody markers of increased risk of malignancy in patients with dermatomyositis. Clin Rev Allergy Immunol.

[10] Ruhlman D, Otten C, Colella C (2014). Dermatomyositis. J Nurse Pract.

[11] Shan DM, Gupta N, Ortega-Loayza AG, Shea S, Nandan A (2024). Anti-nuclear matrix protein 2 antibody-positive amyopathic dermatomyositis presenting in a patient with prostate cancer: a case report. Clin Case Rep.

[12] Aggarwal R, Charles-Schoeman C, Schessl J (2022). Trial of intravenous immune globulin in dermatomyositis. N Engl J Med.

[13] Marie I, Hatron PY, Dominique S, Cherin P, Mouthon L, Menard JF (2011). Short-term and long-term outcomes of interstitial lung disease in polymyositis and dermatomyositis: a series of 107 patients. Arthritis Rheum.

